# Explaining the changes in procrastination in an ACT-based course – psychological flexibility and time and effort management as mediators

**DOI:** 10.3389/fpsyg.2024.1331205

**Published:** 2024-05-01

**Authors:** Henna Asikainen, Telle Hailikari, Nina Katajavuori

**Affiliations:** ^1^HYPE Centre for Teaching and Learning, Faculty of Education, University of Helsinki, Helsinki, Finland; ^2^HAMK Edu Research Unit, Häme University of Applied Sciences, Hämeenlinna, Finland

**Keywords:** procrastination, psychological flexibility, time management, higher education, acceptance and commitment therapy

## Abstract

**Introduction:**

The aim of our study is to explore the relationship between procrastination, time management skills and psychological flexibility and the changes in them during an Acceptance and Commitment therapy (ACT)-based course that included time management training. We also explored the effects of time management skills and psychological flexibility on procrastination. The study used an experimental design in an ACT-based well-being course that included time management training.

**Methods:**

The participants were 109 students taking the course and 27 waiting list students. Analyses were conducted with Pearson correlation, mixed ANOVA and causal mediation analysis.

**Results and discussion:**

Our results show that time management skills, psychological flexibility and procrastination were related to each other, and all changed during the course. In addition, change in both time management and psychological flexibility had an impact on the change in procrastination during the course. The results show that both time management and psychological flexibility influence the change in procrastination during an ACT-based course.

## 1 Introduction

University students experience academic procrastination frequently. Almost all students sometimes procrastinate during their studies, and approximately every second, a student regularly procrastinates (Rothblum et al., 1986; Steel, 2007; Steel and Klingsieck, 2016). Recent research has shown that almost half of students can be classified into having severe procrastination, and of these students, 96% consider procrastination to be a problem (Rozental et al., 2022). In addition, studies have shown that procrastination can lead to many negative consequences. It has been shown to be related to lower academic performance (Steel et al., 2001), increased stress (Sirois et al., 2003) and poorer mental health (Stead et al., 2010) and have consequences for physical and psychological well-being (Rozental et al., 2022). Therefore, it is necessary to understand the causes and ways to affect procrastination.

Many aspects can affect procrastination. Procrastination has traditionally been considered a form of self-regulation failure, as a weakness of will and low ability to organize one’s own studies (e.g., Senécal et al., 1995; Ferrari, 2001; Wolters, 2003; Steel, 2007). This was also found in a recent review study which concluded that most of the studies on procrastination have explored the effect of self-regulation on it (Salguero-Pazos and Reyes-de-Cózar, 2023). One part of self-regulation is time management, and one common theory is that procrastination results from a person’s inability to manage time (Glick and Orsillo, 2015). But time management is only one aspect and does not explain the phenomenon. It has also been suggested that emotional regulation as a part of self-regulation should be taken into account when exploring factors that maintain and cause procrastination. For example, a review study showed that the ability to control and manage emotions is one central aspect studied in procrastination research (Salguero-Pazos and Reyes-de-Cózar, 2023). However, different frameworks of emotional regulation have mainly focused on modifying or regulating the emotions (Salguero-Pazos and Reyes-de-Cózar, 2023). One different way to explore ways to deal with emotions is psychological flexibility (Hayes, 2004; Hayes et al., 2012a), which emphasizes acceptance of emotions instead of changing or controlling them (Hayes et al., 2006). Recent studies concerning procrastination have brought up the importance of psychological flexibility in decreasing procrastination, and research suggests that procrastination results from a person’s psychological inflexibility (Glick et al., 2014; Scent and Boes, 2014; Eisenbeck et al., 2019; Hailikari et al., 2022). However, research has explored different aspects effecting procrastination separately but there is lack of studies exploring different aspects effects together (Salguero-Pazos and Reyes-de-Cózar, 2023). The purpose of this study is to explore the effects of an ACT-based course developing participants’ psychological flexibility and time management skills on procrastination. In addition, we explored the effects of time management and psychological flexibility on procrastination.

## 2 Theoretical background

Procrastination may be defined as ‘the voluntary delay of an intended and necessary and/or [personally] important activity, despite expecting potential negative consequences that outweigh the positive consequences of the delay’ (Klingsieck, 2013, p. 26). Procrastination may be defined as self-handicapping behavior that occurs when a person postpones a task they intend to complete, potentially leading to increased stress (Steel, 2007). Procrastination is often conceptualized as a self-regulation failure, meaning that there is a gap between an individual’s intentions and actions (Dewitte and Lens, 2000) rather than an intention to delay. Typical for procrastination is that it is needless, counterproductive, and accompanied by feelings of discomfort (Schraw et al., 2007). Klingsieck (2013) further claims that typically procrastination is irrational, and people cannot control their procrastination even if they want to (see also Grunschel and Schopenhauer, 2015).

Most of the studies on procrastination have focused on academic procrastination – that is, procrastination of study-relevant activities of university students (van Eerde and Klingsieck, 2018; Salguero-Pazos and Reyes-de-Cózar, 2023). In the academic context, procrastination is often associated with several negative factors, such as lower academic performance (Steel et al., 2001), increased stress (Sirois et al., 2003), poorer well-being (Kim and Seo, 2015) and poorer mental health (Stead et al., 2010). There is no single factor that could fully explain the reason for procrastination. There are several different theoretical approaches providing explanations for procrastination behavior (Klingsieck, 2013; Salguero-Pazos and Reyes-de-Cózar, 2023). In the academic context, individual motivational factors, such as motivation, self-regulation, time management and learning strategies, are the most relevant, as these factors may be addressed by pedagogical choices. Some of the other approaches focus more on, for example, personality traits and disorders, which are not so easily influenced by pedagogical choices but rather require more psychological expertise.

One different way to explore ways to deal with emotions is psychological flexibility (Hayes, 2004; Hayes et al., 2012a), which emphasizes acceptance of emotions instead of changing or controlling them (Hayes et al., 2006). Recent studies concerning procrastination have brought up the importance of psychological flexibility in decreasing procrastination, and research suggests that procrastination results from a person’s psychological inflexibility (Glick et al., 2014; Scent and Boes, 2014; Eisenbeck et al., 2019; Hailikari et al., 2022). This highlights the need to develop skills in psychological flexibility to decrease procrastination: important is to develop one’s skills to accept negative and difficult feelings and emotions which may arise in difficult learning situations; and further, to take actions – meaning get to work – despite these difficult feelings.

### 2.1 Time management affecting procrastination

Procrastination is frequently viewed as a failure in self-regulation, characterized by a lack of willpower and an inability to organize one’s own studying (Wolters and Brady, 2021). Recent studies suggest that procrastination often stems from an individual’s struggle to effectively manage their time (Hailikari et al., 2021; Fentaw et al., 2022; Sefriani et al., 2022). Time and effort management skills encompass a university student’s capacity to establish goals, study in alignment with those goals, manage their time efficiently and prioritize tasks (Entwistle, 2001). Studies have consistently demonstrated that these skills are crucial factors in promoting academic progress (e.g., Pintrich, 2004; Haarala-Muhonen et al., 2011; Rytkönen et al., 2012; Häfner et al., 2015; Asikainen et al., 2020). Furthermore, it has been shown that many higher education students encounter challenges in developing and enhancing time management skills during their studies (Parpala et al., 2017).

Time management has been found to be related to procrastination. It has been shown that poor time management skills are positively related to procrastination behavior (Wolters et al., 2017; Fentaw et al., 2022). In a study by Wolters et al. (2017), time management skills had the strongest association with procrastination compared to other aspects such as motivation and metacognition factors. It also has been shown that procrastination can be decreased with time management training (Häfner et al., 2014), and online studying, which has been increasing in university studies since the pandemic, can cause time management problems. Additionally, students’ beliefs about their self-regulation skills, such as time management, are related to procrastination (Han et al., 2023).

People who fail to self-regulate their own behavior, especially when facing aversive tasks, often give into the short-term rewards and instant gratification that can come when avoiding a task (Sirois and Pychyl, 2013). Thus, it is a matter of deficit in emotional regulation over long-term goals. Recent research suggests that, instead of being purely a self-regulation or time management problem, procrastination is strongly influenced by an inability to cope with negative emotions that arise in challenging situations (Gagnon et al., 2016; Hailikari et al., 2021). One promising and effective skill for emotional regulation is psychological flexibility, and thus, it is necessary to consider it as a central factor influencing procrastination.

### 2.2 Psychological flexibility

One aspect affecting students’ procrastination is psychological flexibility (Glick et al., 2014; Sutcliffe et al., 2019). Psychological flexibility describes people’s ability to direct their behavior and attention towards actions that are meaningful for them flexibly in the presence of negative thoughts and feelings (Hayes et al., 2006; Chawla and Ostafin, 2007). Thus, people with high psychological flexibility can function according to what is really in line with their values or meaningful for them and by living a value-based life, accepting negative thoughts, emotions and sensations by taking on an observer perspective to them and opening up to them mindfully (Bond et al., 2010). The origin of the concept comes from Acceptance and Commitment Therapy (ACT), which is one of the third-wave therapies focused on reducing *experiential avoidance,* which is an opposite process of psychological flexibility and can be regarded as psychological inflexibility (Hayes et al., 2006). Experiential avoidance means avoiding negative feelings, sensations, or thoughts in a way that it causes harm in the long run (Hayes et al., 2012a).

Psychological flexibility realizes though six overlapping processes: cognitive defusion, being present, self as context, acceptance, values and committed action (Hayes et al., 2006). Cognitive defusion is a core process underlying the third-wave therapies and represents the process where one learns to look at one’s thoughts from an observer’s perspective rather than letting the thoughts define their actions (Hayes, 2019). Cognitive defusion thus means that one can look at feelings and thoughts as separate and not consider them as truths about oneself (Hayes, 2019). Being present is an important process for cognitive defusion, as it means being able to focus on the present moment instead of living in the past or future and, thus, being in contact with events or thoughts as they occur, emphasizing noticing them as an observer instead of being judgmental towards them (Hayes et al., 2006). Being present is closely related to seeing oneself as a context or a container of emotions instead of being the emotions (Hayes, 2019). That is to say, self as context includes seeing oneself as an observing self who is separate from the emotions and thoughts (Hayes et al., 2012c). Acceptance is the opposite of avoiding and running away from or fighting against difficult emotions and thoughts but instead actively accepting difficult emotions as they come without trying to change them (Hayes et al., 2006).

Living a meaningful life is a core aim with developing psychological flexibility. Shifting the focus from short-term rewards involves really exploring one’s values and what is important to oneself. It is an important part of the development of psychological flexibility (Hayes et al., 2006). People often can have different kinds of aims that they think they must do, such as for social acceptance, and do not really think about what is truly meaningful for themselves (Hayes, 2019). The sixth aspect of flexibility, committed action, means that people take actions based on their values and what is meaningful for them with the help of the other five processes (Hayes et al., 2006).

A total of 40 years of research has shown that ACT intervention targeted at increasing psychological flexibility has an important impact on improving well-being and life satisfaction as well as decreasing a wide range of psychological problems such as depression, anxiety and stress for different populations including university students (for review studies and meta-analyses, see Howell and Passmore, 2019; Bai et al., 2020; Gloster et al., 2020). Furthermore, the importance of psychological flexibility has been noticed in relation to university studies. It has been shown that psychological flexibility is positively related to positive emotions and progression in studying (Asikainen et al., 2018; Hailikari et al., 2022). In addition, it is positively related to self-regulated learning (Asikainen et al., 2018) and integration in studies (Asikainen, 2018) and negatively related to self-handicapping strategies in studies (Hailikari et al., 2022). These self-handicapping processes have been shown be related to psychological inflexibility, as studies have shown that psychological inflexibility such as avoiding negative emotions and thoughts is clearly related to experiences of procrastination (Dionne, 2016; Gagnon et al., 2016, 2019). In a study by Eisenbeck et al. (2019) they found that psychological inflexibility mediated between procrastination and several aspects comprising psychological distress, anxiety, depression and stress. They argued that psychological inflexibility is an underlying mechanism of procrastination as procrastination may result from not procrastinating students not only experiencing negative emotions but avoiding them (Eisenbeck et al., 2019). ACT-based interventions promoting psychological flexibility have been shown to decrease procrastination (Glick et al., 2014; Gagnon et al., 2016, 2019). and to give longer-term effects than for example cognitive behavioral therapy-based interventions (Wang et al., 2017). Furthermore, it has been suggested that approaches to increase psychological flexibility in order to reduce procrastination and enhance students’ wellbeing should be explored (Sutcliffe et al., 2019). Thus, it is fair to suggest that the core aspect in psychological flexibility which is to make choices to act based on what is meaningful despite of the possible discomfort it may cause by accepting these negative feelings and thoughts is one of the main processes that could also effect procrastination as it includes the unnecessary postponement of activities which are related to a goal (Knaus, 2000).

## 3 Research questions

Time management training as well as ACT-based training can have effects on procrastination, but few studies have explored these effects together. One study has shown that both psychological flexibility and time management skills explain procrastination (Hailikari et al., 2022), but the study was done at one timepoint. The aim of this study is to explore the relationship between procrastination, time management and psychological flexibility and the changes in them during an ACT-based course that includes time management training. We also explore the effects of time management skills and psychological flexibility on procrastination. Specific research questions are:

- How is procrastination related to psychological flexibility and time management?
- How does the ACT-based course affect students’ psychological flexibility, procrastination and time management?
- How do changes in psychological flexibility and time management mediate the effect between the course and changes in procrastination?

We expect that time management skills and psychological flexibility are related to procrastination based on previous research (Glick et al., 2014; Gagnon et al., 2016; Hailikari et al., 2021). We expect that the ACT-based course, which includes time management training, has an impact on students’ time management skills (Häfner et al., 2015), psychological flexibility (Räsänen et al., 2016) and procrastination (Gagnon et al., 2016). In addition, we expect that both psychological flexibility and time management skills have an effect on procrastination during the course (Hailikari et al., 2021; Katajavuori et al., 2021).

## 4 Method

### 4.1 Participants

The data were collected as part of an optional eight-week online course aiming to foster students’ well-being and studying. The course was organized in the fall of 2021 at the University of x, and students could get 3 ECTS for completing the course. The course was based on developing students’ psychological flexibility processes as well as study skills such as time management. The course included seven different themes comprising introduction materials, individual exercises and group discussion on a weekly basis. The themes included, for example, reflecting on one’s values, self-compassion, defusion, mindfulness and committed action (see Table 1). In addition, students did a time management exercise in which they were asked to monitor and record their time management for a week and reflect on what they learned from it. Students wrote a reflective learning report at the end of the course, reflecting on the course and how it affected their studying. Completing the course required submitting the assignments on time and participating to peer group meetings, and course was assessed on a pass-fail basis. The course was delivered and developed by the university lecturers working in the Centre × (reference).

**Table 1:** The themes of the course.

**Week**	**Course themes**
1	Orientation to the course: familiarizing with the concept of psychological flexibility, assessing one’s own well-being and setting goals for the course. Time usage exercise and reflection.
2	Values: learning about values and their meaning for one’s well-being, recognizing and reflecting on one’s own values and strengths.
3	Relaxation and concentration: learning about stress and mindfulness and learning mindful exercises that can help to reduce stress.
4	Thoughts and cognitive defusion: learning about defusion and its connection to well-being and procrastination. Exercising defusion.
5	Lifestyle skills and learning techniques (committed action): how to live a healthy life, taking into account sleep, exercise and nutrition. Learning different learning strategies.
6	Self-compassion: learning about self-compassion and its connection to well-being. Different exercises to support self-compassion.
7	Values-based life (committed action): practicing committed actions and a flexible self-concept. Learning to live a life according to values by observing goals, choices and actions.
8	Feedback section: giving peer feedback on learning reports and giving course feedback.

The study was conducted in line with the Ethical principles of the Declaration of Helsinki. The participants were informed about the research. Filling in the questionnaire data was part of the course assignment where they filled out the questionnaires and got feedback on them in the beginning and at the end of the course but participating in the research was voluntary and a consent was collected for using their data in our research. Participation in the study did not affect course completion in any way, and students had the possibility to change their consent to participate in the study at any stage during the course. A total of 151 students completed the course. Of these 151 students, 109 answered both questionnaires in the beginning and at the end of the course and gave permission to use their answers in the research. Of these students, 94 were female and 15 were male. A total of 37% of the students were first-year students, 28% were second-year students, 16% were third-year students, 8% were fourth-year students and the rest started their studies before 2018. The ages ranged between 20 and 60 years (mean = 26.97, median = 24.99, Std = 7.08). The control group comprised 27 students on the waiting list to another similar course in the spring of 2022. Of these students, 23 were female and 4 were male. Total of 48% of the students were first-year students, 7% were second-year students, 11% were third-year students, 15% were fourth-year students and the rest started their studies before 2018. The ages ranged between 20 and 52 years (mean = 32.37, median = 28.46, Std = 10.12). These students in control group completed the questionnaires at the same time than the students who participated to the course. The students were from different faculties and disciplines from the university, they did not know each other and were not in contact during the course.

### 4.2 Instruments

Time and effort management was measured with the organized studying scale, which includes statements concerning students’ time and effort management behavior (four items) from the HowULearn questionnaire (Parpala and Lindblom-Ylänne, 2012). Its scales are widely used and validated in Finnish and international contexts (e.g., Parpala et al., 2010; Rytkönen et al., 2012; Ruohoniemi et al., 2017; Postareff et al., 2018; Cheung et al., 2020). Time and effort management skills were measured with four items on a Likert scale from 1 = totally disagree to 5 = totally agree (e.g., ‘I am generally systematic and organized in my studies’). Psychological flexibility was measured with the compact questionnaire (Francis et al., 2016), which has been widely used in different contexts (Tatta et al., 2022; Eadeh et al., 2023; Zhao et al., 2024). The items used a seven-point Likert scale (0 = strongly disagree to 6 = strongly agree). Procrastination was measured with a short version of the pure procrastination scale (PPS) (Svartdal and Steel, 2017) using a five-point Likert scale (five items, e.g., ‘In preparation for some deadlines, I often waste time by doing other things’). This short version of the original PPS has been proven to be a robust instrument to measure academic procrastination (Svartdal and Steel, 2017).

### 4.3 Analysis

The relationship between psychological flexibility, procrastination and time and effort management was analyzed with the Pearson correlation. The change in psychological flexibility, time and effort management and procrastination was analyzed with a mixed ANOVA comparing the experimental and control group using the Time × group association. Change variables of the scales measuring procrastination, time management and psychological flexibility were conducted by subtracting the sum of the first measurement from the second measurement. The effects of the course on change in procrastination with psychological flexibility and organized studying as mediators was conducted with causal mediation analysis using Spss Process.

## 5 Results

The correlational analysis showed that procrastination correlated negatively with time and effort management in the first (*r* = −674, *p* < 0.001) and second (*r* = −0.654, *p* < 0.001) measurements. A negative correlation between psychological flexibility and procrastination was found in the first (−0.394, *p* < 0.001) and second (−0.459, *p* < 0.001) measurements. In addition, time and effort management and psychological flexibility correlated positively with each other in the first (*r* = 0.398, *p* < 0.001) and second (*r* = 0.377, *p* < 0.001) measurements. Results of the correlation analysis can be seen in Table 2.

**Table 2:** Correlations between procrastination, organized studying and psychological flexibility in the beginning and at the end of the course.

	PR1	PR2	OR1	OR2	PF2	PF2
PR1 Procrastination 1	1					
PR2 Procrastination 2	0.781***	1				
OR1 Organized 1	−0.674***	−0.516***	1			
OR2 Organized 2	−0.620***	−0.657***	0.695***	1		
PF1 Psychological flexibility 1	−0.394***	−0.283***	0.398***	0.302***	1	
PF2 Psychological flexibility 2	−0.386***	−0.459***	0.300***	0.377***	0.690***	1

***p < 0.001.

The mixed ANOVA showed that time and effort management and psychological flexibility increased statistically significantly (Time × group) compared to the control group (*p* = 0.011–0.030). In addition, procrastination decreased statistically significantly compared to the control group (*p* = 0.015). The results from the mixed ANOVA analysis can be seen in Table 3.

**Table 3:** Results of the mixed ANOVA analysis.

			Time		Group		Time × group	
	M1(sd)	M2(sd)	*F*	*p*-value	*F*	*p*-value	*F*	*p*-value
Time management	3.19 (0.96)	3.45 (0.81)	4.305	0.040	3.935	0.049	4.835	0.030
Psychological flexibility	3.55 (0.88)	3.95 (0.83)	15.650	<0.001	0.235	0.628	6.604	0.011
Procrastination	3.30 (1.13)	2.91 (1.12)	12.351	<0.001	1.855	0.176	6.128	0.015

**Figure 1:**
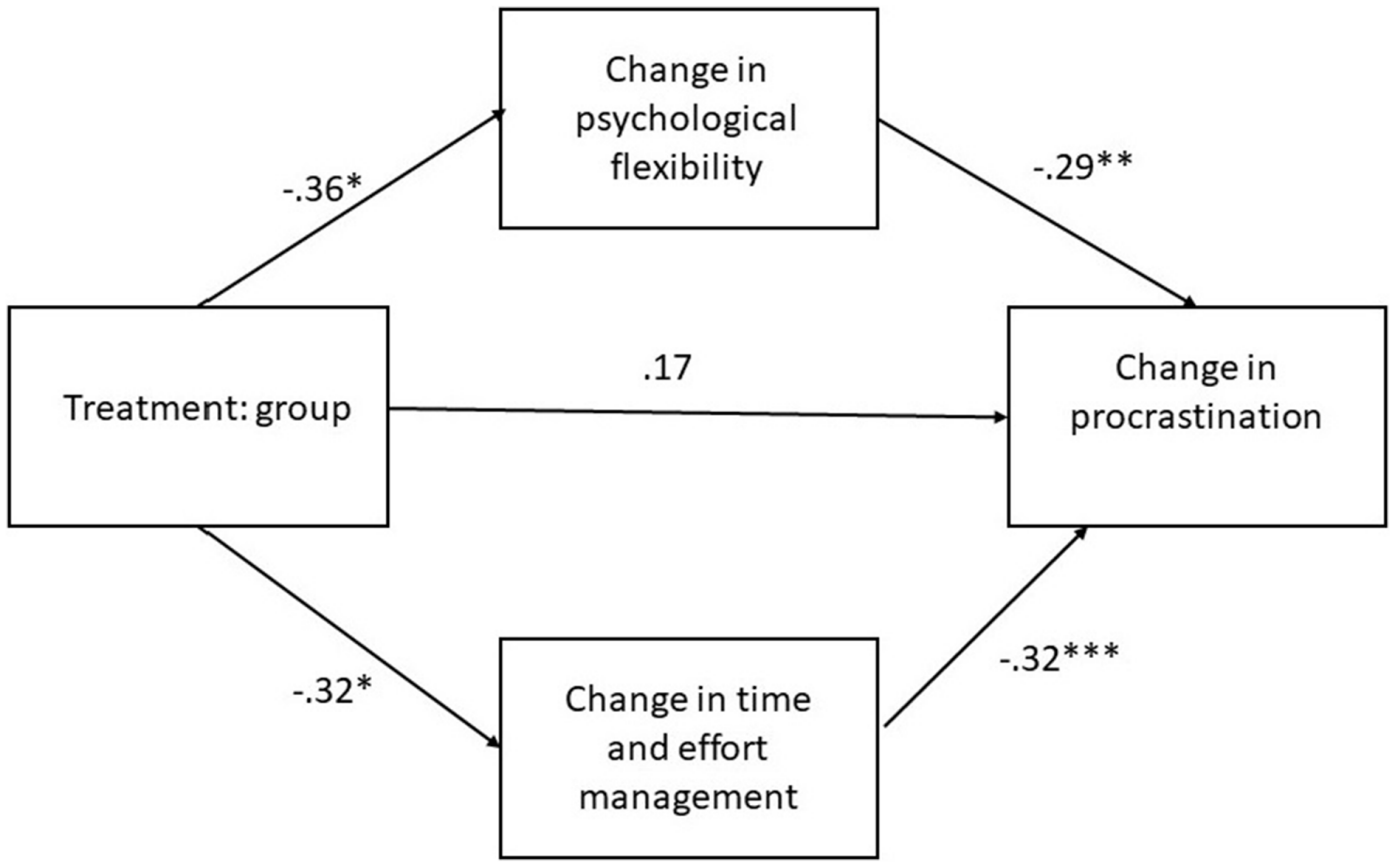
The effects on the course on change in procrastination through mediating variables change in psychological flexibility and time and effort management.

The Hayes process Macro model 4 was applied to assess the direct, indirect and total effects between the treatment variable group and outcome variable change in procrastination with change in psychological flexibility and time and effort management as mediating variables. The analysis showed that the treatment variable group has statistically significant direct effects on the change in time and effort management (*p* = 0.030) and change in psychological flexibility (*p* = 0.011). The total effect of the model was 0.213 and the indirect effects from treatment to changes in procrastination through both change in psychological flexibility and time and effort management were statistical significant (*p* < 0.005) (see Figure 1).

## 6 Discussion

The aim of this study was to explore the relationship between procrastination, time and effort management and psychological flexibility and changes in them during an ACT-based course. We also explore the effects of change in time and effort management and psychological flexibility on procrastination.

The results of our study showed that time and effort management and psychological flexibility were both negatively related with procrastination. This finding is in line with previous studies that have shown that time and effort management skills and psychological flexibility are both associated with procrastination (Ferrari, 2001; Wolters, 2003; Steel, 2007; Glick et al., 2014; Dionne, 2016; Gagnon et al., 2016, 2019; Hailikari et al., 2021). Furthermore, the results of our study showed that there was a correlation between psychological flexibility and time and effort management skills. This finding is in line with previous studies that have found a negative correlation between psychological flexibility and time management (Asikainen et al., 2019; Hailikari et al., 2022). In a study exploring profiles based on time management and psychological flexibility by Hailikari et al. (2022), it was suggested that time management skills and psychological flexibility skills go hand in hand. In our course setting, both skills were supported, but a clear correlation was found both in the beginning and at the end of the course. Previous studies have shown that psychological flexibility is related to better self-regulation skills and better progression in studies (Asikainen et al., 2018; Eisenbeck et al., 2019; LeJeune and Luoma, 2019; Hailikari et al., 2022); it is also positively related to time and effort management skills (Hailikari et al., 2022).

The results of our study showed that, during an online ACT-based course that included time management skills, and psychological flexibility increased and procrastination decreased compared to the control group. Previous studies have shown that ACT-based interventions can increase psychological flexibility skills (e.g., Räsänen et al., 2016). This was evident in our online course, in which the group served as support for the individuals, and the teacher had a very small role. Previous studies have shown that peer-group-supported ACT-based interventions can support psychological flexibility (Grégoire et al., 2022), but in this study, the peers were trained to lead the intervention. It seems that self-directed group discussion has an impact as well. In addition, previous studies have shown that time management skills can be increased by practicing with interventions (Häfner et al., 2014). Although the time management training in this course centered on time usage follow-up for 1 week and reflection of time usage, it seems that it was enough to make an impact during the course. The positive and long-term effects of time management training have been found in earlier studies (Green and Skinner, 2005; Wingren et al., 2022), but our study showed that the effects were reached already after a one-week follow-up. Time usage follow-up can add students’ perceived control over time, which has been found to be related to time management and well-being (Chang and Nguyen, 2011).

In addition, there was impact on procrastination during the course, which was expected, as it has been shown that both time-management-based (Häfner et al., 2014) and ACT-based interventions (Gagnon et al., 2016) can decrease procrastination. The results suggest that, with an ACT-based course including time usage follow-up and reflection, it is possible to decrease students’ experiences of procrastination in their studies. Furthermore, the results of our study showed that both changes in time management and psychological flexibility have an impact on change in procrastination in this course. This indicates that reducing procrastination can be done not only by promoting time management skills but also by focusing on developing psychological flexibility skills. Psychological flexibility promotes value-based actions, despite all the negative emotions and feelings one might have, and decreases avoidance behavior (Hayes et al., 2006). Thus, psychological flexibility can help students’ time management by giving them tools to allow time for important aspects of life and to commit to one’s value-based goals.

The role of psychological flexibility in procrastination has also been shown in previous studies. It has been stated that procrastination may include the decreased ability to be present (Glick et al., 2014), which is a central part of psychological flexibility (Hayes et al., 2006). Previous studies have also shown that, for example, perfectionism, including a very critical assessment of one’s own behavior and performance, which is evident among university students, is related to procrastination (Çapan, 2010; Ashraf et al., 2023). Psychological flexibility skills help one to accept difficult emotions and thoughts about oneself and can help to develop a less critical and more lenient attitude towards oneself, living life according to one’s own values instead of avoidance (Hayes, 2019). This acceptance of difficult emotions and value-based actions instead of experimental avoidance are key aspects of psychological flexibility and are shown to be in the heart of procrastination, which is a problem of avoidance (Dionne and Duckworth, 2011).

### 6.1 Limitations

There are some limitations that need to be taken into account concerning this study. Even though this course was optional for all the students in the university, it is likely that this study included a selected sample of students who were interested in improving their well-being and studying because this course was advertised as a course that especially aims to foster participants’ well-being. Thus, the generalization of the results should be considered carefully. The number of the participants could have been higher, and only two-thirds of the students who participated in this course participated in this research. It is possible that the group of participating students differed from those who did not give their consent form. In addition, the number of the students in the control group was very small. Unfortunately, we were unable to get more answers from the waiting list participants. This could have had an impact on the study results. However, there is evidence that reliable results can be obtained even though the control group is much smaller (Hutchins et al., 2021) Furthermore, this study did not include a follow-up measurement; thus, it is not possible to say how permanent the changes concerning procrastination and the other measurements were. In the future, it is important to have a longitudinal setting to explore how permanent the benefits that can be gained during this intervention course are. In addition, future research should consider how the course affects study progression measured with earned credits. The present study included only self-reported measures.

### 6.2 Practical implications

In addition to improving students’ well-being and studying in several ways (Asikainen et al., 2019; Katajavuori et al., 2021), an ACT-based intervention combined with time management training in the higher education context can be a beneficial way to diminish procrastination in the academic context. These kinds of courses are able to improve time management and psychological flexibility skills, which can be considered very important future working life skills as well. Different kinds of interventions have been delivered to students as part of counselling, but these kinds of courses could be implemented in curriculums. Furthermore, the skills for both time and effort management and psychological flexibility should be supported during higher education. It would be important that pedagogical awareness and pedagogical skills of the teachers would improve, and that practical teaching work would include guidance on breaking down goals and tasks. Furthermore, including formative assessment to teaching would make it possible for students to get feedback of their progression of studies and their performance and that way, help the students in their effort management. The teachers could also make it explicit that learning and completing the course may not always be nice but may include difficult feelings and emotions, which do not disappear by avoiding the assignments and are part of normal life and studying.

In addition, New approaches and ways to reduce procrastination has been called for (Goroshit, 2018; Zacks and Hen, 2018), and one suggestion to enhance student well-being and reduce procrastination is to increase psychological flexibility among university students (Sutcliffe et al., 2019). Based on our study, it seems that this kind of online intervention course could be one way to accomplish this.

## Data availability statement

The raw data supporting the conclusions of this article will be made available by the authors, without undue reservation.

## Ethics statement

Ethical review and approval was not required for the study on human participants in accordance with the local legislation and institutional requirements. The studies were conducted in accordance with the local legislation and institutional requirements. The participants provided their written informed consent to participate in this study.

## Author contributions

HA: Methodology, Writing – original draft, Writing – review & editing. TH: Writing – original draft, Writing – review & editing. NK: Writing – original draft, Writing – review & editing.
